# A geochemical and mineralogical characterization of soils associated with podoconiosis

**DOI:** 10.1007/s10653-023-01625-5

**Published:** 2023-07-15

**Authors:** Jamey N. Cooper, Kevin E. Nick

**Affiliations:** Department of Earth and Biological Sciences, Loma Linda University, Loma Linda, CA 92350 USA

**Keywords:** Ethiopia, Weathering, Multivariate statistics, Etiology, Tropical disease, Silica

## Abstract

Podoconiosis is a disease that causes swelling and disfiguration of the lower legs found in several developing countries where shoes are not regularly worn. The current model for the etiology of the disease proposes that mineralogical agents enter the lymph system through the skin leading to inflammation that causes swelling of the feet and legs. We collected 125 soil samples from 21 towns associated with podoconiosis, 8 towns unassociated with Podoconiosis as controls, and 3 towns of unknown status. Data collected for each soil sample included color, particle size, mineralogy, and geochemistry to distinguish unique components within the podoconiosis-associated soils. Our results indicate podoconiosis-associated soils are more highly weathered than non-podoconiosis associated soils. The enrichment of kaolinite and gibbsite suggests that these minerals, their surface chemistry, and trace elements associated with them should be prioritized in future podoconiosis research. In addition, we found that color may be a valuable tool to identify soils at greater risk for inducing podoconiosis.

## Introduction

Podoconiosis is a disease that causes swelling and disfiguration of the lower legs affecting individuals living on or around specific volcanic areas. Worldwide, podoconiosis affects about four million people in at least 32 countries, with a significant incidence in Africa, South America, and the Indian subcontinent. Ethiopia has the unfortunate distinction of being the country most highly affected, with geostatistical modeling estimates of 1,539,963 affected individuals in 2015 (Davey et al., 2007; Deribe et al., 2017). The last decade has seen a significant increase in studies of Podoconiosis. Improvements are occurring in the understanding of its medical and public health aspects, such as prevention and management, genetic factors, and even community understanding (Alcantara et al., 2020; Davey et al., 2007; Deribe et al., 2018; Florence & Fuller, 2017; Gebresilase et al., 2021; Molla et al., 2012; Tora et al., 2016; van‘t Noordende et al., 2020). However, research into the soils has not received the same attention, though recent years suggest growing interest (Cooper et al., 2019; Gislam et al., 2020; Le Blond et al., 2015; Molla et al., 2014).

Literature on Podoconiosis postulates that some compositional or textural fractions of certain tropical soil types are causal factors for this disfiguring disease. However, despite decades of study, no causal connection between the disease and a specific soil fraction has been identified. Therefore, the effects of a particular soil cannot be predicted or mediated for this disease. Since the minerals, elements, and grain sizes suspected in the proposed etiology are common in many soils, but the disease is isolated geographically, a need exists to determine if unique compositions, textures, or mineral suites are present in podoconiosis-associated soils. This paper adds to the growing geochemical, textural, and mineralogical data available for soils associated with Podoconiosis and examines correlations with disease occurrence. Our goal was to gather a widely distributed set of samples and systematically test the current geological hypotheses for the etiology of podoconiosis.

## Mineralogical and geochemical background

The etiology of podoconiosis is still unclear, but as far back as the 1800s, a correlation between podoconiosis and the environment led to theories that some components of the local soil were causative agents. Disease occurrence suggests enrichment of a particular phase due to specific bedrock geochemistry underlying endemic locations, unique weathering products during soil formation, or both (Cooper et al., 2019; Gislam et al., 2020). The association between volcanic soils and specific professions directly and frequently in contact with soil strengthened the mineral-related hypothesis. Agricultural workers are frequent examples as they work daily in fields where bare feet are in constant contact with the soil, thus with the particles believed to induce podoconiosis (Price & Bailey, 1984; Wanji et al., 2021).

Research over the years has sought to determine whether the association between soil and disease could be demonstrated, and several theories for the soil's toxicity have been proposed. The first major hypothesis, formulated by Heather and Price (1972), identified particles containing silicon, aluminum, and iron inside the lymph nodes of individuals living in regions both affected and unaffected by podoconiosis. The presence of silicon and aluminum, in particular, have been the focus of many studies attempting to identify statistical differences in their presence between soil groups. This research led to the identification of quartz or silica as the causal organisms for podoconiosis for several decades, but more recent research by Cooper et al. (2019) and Gislam et al. (2020) has noted that quartz is an unlikely candidate due to its ubiquity and inert chemistry. This geologic connection, however, led to Price's first market count, which confirmed a higher percentage of disease in those spending more time on the red soil, which contained kaolinite and montmorillonite (Price, 1974). This data introduced the second line of inquiry focused on the specific clay species present in the soils and hypothesizing that adsorption of trace elements may lead to toxicity within the bodies of individuals who are in regular contact with these soils. The final major area of research for podoconiosis has focused on the significance of particle size. Price (1974) hypothesized that colloid-size particles might be etiologically significant as they can more easily move through microtraumas of the feet. Though several studies have looked into this, results are conflicting, indicating that size may not be a key factor.

Of course, work on identifying mineralogical or geochemical agents is merely the first step. Any component believed to be indicated in the disease must also be tested for the immunological response expected from a causal agent. The only publication on this subject to date is Le Blond et al. (2017), who reported that kaolinite, smectite, and quartz elicited a stronger immune response than other mineral phases tested but found no large difference in hemolytic activity between the soils from endemic and non-endemic areas.

## Methods

### Soil collection

**Fig. 1:**
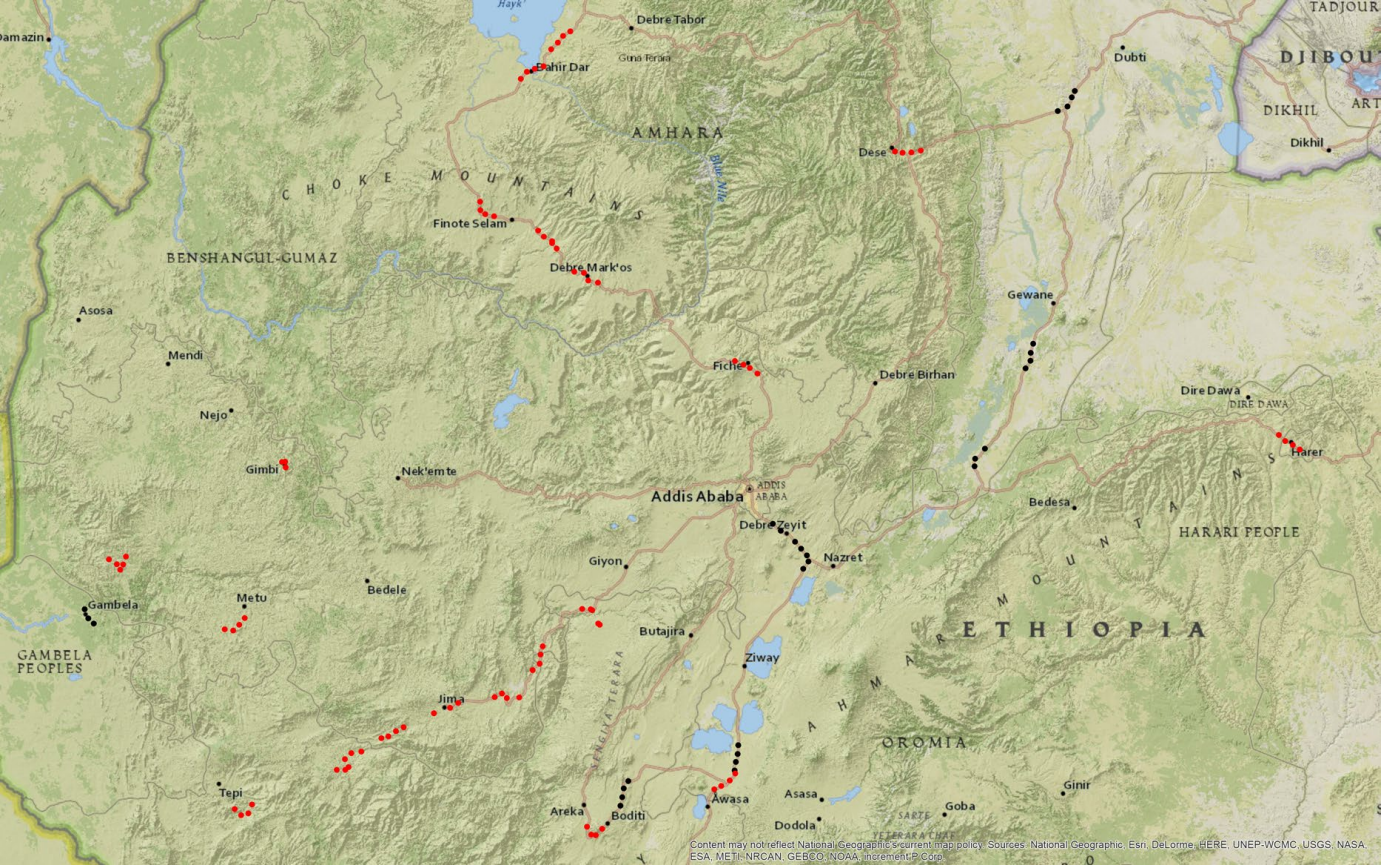
Map of Ethiopia showing sample locations (n = 125) including podoconiosis-associated (red) and non-podoconiosis associated (black). Sample locations with unverified disease status used as test cases are not plotted

A total of 125 samples were collected from 32 towns (21 podoconiosis-associated (PAS), 8 non-podoconiosis-associated (non-PAS), from previously published locations, and 3 of unknown disease status) across Ethiopia (Fig. 1; Table 1). Samples were collected at 3 and 6 km on either side of the town center. Near the roadside, the top few cm of soil were removed, and a 50 ml sample was collected from the upper 12 inches. Samples were placed directly into plastic collection tubes with screw caps and GPS tagged.

**Table 1:** Sampling data including town, region, number of samples collected, reference citation, disease prevalence and GPS coordinates (*n* = 125)

Town, region	Citation	Prevalence (%/index/index)	Latitude, longitude
*Disease present (21 towns/84 samples)*			
Asendabo, Oromia	1	1.20	07.773738, 37.227645
Bahir Dar, Amhara	1/2	2.85/1.85	11.574208, 37.361353
Bonga, SNNPR	1	3.06	07.267190, 36.246813
Bure, Amhara	1	6.31	10.706105, 37.066865
Debre Markos, Amhara	1/2	2.42/2.32	10.329634, 37.734399
Dembecha, Amhara	1	3.76	10.562716, 37.494811
Dembi Dolo, Oromia	1/5	1.06	08.533310, 34.801695
Fiche, Oromia	1, 2	1.80/1.58	09.785285, 38.731582
Ghimbo, SNNPR	1	2.21	07.397408, 36.231472
Gimbi, Oromia	1, 2	2.39/3.56	09.186040, 35.833449
Gore, Oromia	1, 5	6.87	08.151342, 35.535687
Hamusit, Amhara	1	1.99	11.783043, 37.561445
Harar, Harari	1	4.40	09.312556, 42.122682
Jimma, Oromia	1	1.73	07.673891, 36.835793
Kombolcha, Amhara	1	0.90	11.084934, 39.729184
Mizan Teferi, SNNPR	1	4.37	06.993647, 35.582199
Saja, SNNPR	1	1.95	07.974374, 37.439209
Shebe, Oromia	1	3.23	07.506283, 36.513505
Sheshamane, Oromia	1, 2	1.02/1.10	07.197297, 38.600533
Sodo, SNNPR	1, 3, 4	8.79/5.38/6.18	06.852809, 37.760969
Welkite, SNNPR	1, 2	0.69/2.41	08.289729, 37.782218
*Disease not present (8 towns/29 samples)*			
Debre Zeyit, Oromia	5		08.744749, 38.986477
Debu, Afar	5		09.240414, 40.144654
Gambella, Gambella	5		08.247190, 34.591597
Maderala Dala, Afar	5		09.833333, 40.516667
Mile, Afar	5		11.422231, 40.763999
Mojo, Oromia	5		08.587018, 39.123146
Negele, Oromia	5		07.361089, 38.668711
Shone, SNNPR	5		07.139832, 37.954621
*Unknown (3 towns/12 samples)*			
BAB			
BUL			
GOW			

References are coded as follows: 1 = Oomen 1969, 2 = Price 1973, 3 = Price 1974a, 4 = Price 1974b, 5 = Price 1976. Latitude and longitude provided are for the town center (WGS84 datum)

### Color analysis

All 113 samples were measured using the Anaheim Scientific H500 RGB color analyzer (Yorba Linda, CA). Dry samples of approximately 30–40 g were placed to completely cover the bottom of weighing boats, and spectra were collected while isolated from ambient light. Five measurements were taken for each sample. The five measurements from individual samples were averaged, and all samples from a single town were also averaged for a single value representing each town.

### Particle size analysis

For each sample, 1 g of soil was suspended in 7.37 mM Na_2_CO_3_. This buffer showed the maximum dispersive potential based on zeta potential measurements. The suspension was placed in a 50 ml tube and sonicated for 15 min at 40 kHz using a Branson 1510 with a 0.25-inch tip (Danbury, CT). Grain size measurements used a Beckman Coulter LS13 320 (Indianapolis, IN), with readings over 120 s, pump speed of 25%, and obscuration of 3%. Data collected included mean, median, and modes and specific size fraction ranges: weight % < 1.0 µm (colloid), 1–3.89 µm (clay), 3.9–62.9 µm (silt), and > 62.9 µm (sand). We also analyzed data for the < 10 µm fraction as most macrophages (the cells that would engulf the particles should they enter the body) could easily engulf this size. Data from individual samples were averaged together and used as a single value for each town.

A note about the use of the word "clay" in this paper: It can become confusing when the word clay is used as it can mean both the particle size category and the mineralogical component, such as kaolinite or smectite. We will use the clarifier "clay-sized” or “particle size" when discussing a size fraction and "clay" when discussing the mineral.

### Mineralogical Analysis

Bulk samples were packed using the rear-loading technique and measured in a Bruker D8 Advance X-ray diffractometer (Madison, WI) with CuKα radiation ($\lambda$ = 1.5406 Å). Data were collected continuously from 5–65° 2*θ* binned to a step size of 0.02° at 40 kV and 40 mA. Diffraction data were analyzed with Jade software (Materials Data Incorporated; Livermore, CA; 2010). After analysis, all minerals identified were grouped into 7 major groups: carbonate, clay, feldspar, mica, oxyhydroxide, pyroxene, and quartz for final analysis.

Oriented clay mounts were prepared following Moore and Reynolds Jr (1989). 1 g of soil was sonicated in 7.37 mM Na_2_CO_3_ for 5 min in a water bath. Mixture was then alloquoted and centrifuged at 2000 rpm for 5 min. Supernatant was vacuum filtered onto 0.045 µm HA nitrocellulose membrane filter paper (MilliporeSigma; Burlington, MA) and then transferred to glass slides. Data were collected continuously from 2° to 30° 2*θ* binned to a step size of 0.02° at a voltage of 40 kV and current of 40 mA. Measurements were repeated after ethylene glycol solvation to investigate the presence of swelling clays. Analysis was performed using Newmod software (Crofton, MD). After analysis, all clays identified were grouped into 3 major groups: dimica, kaolinite, and smectite for final analysis.

### Geochemical analysis

Composite samples (21 PAS, 8 non-PAS, and 3 of unknown disease status) were sent to external labs (Actlabs, Ancaster, Ontario (29 samples: 9 PAS, 7 non-PAS, 3 replicates) and SGS, Burnaby, British Columbia (100 samples: 19 PAS, 7 non-PAS, 3 unknown) for analysis of major and trace element composition using ICP-MS/AES. Analyses gave ten major elements as weight percent oxides (Al_2_O_3_, CaO, Fe_2_O_3_, K_2_O, MgO, MnO, Na_2_O, P_2_O_5_, SiO_2_, and TiO_2_) and 47 trace elements (Ag, As, Ba, Be, Bi, Cd, Ce, Co, Cr, Cs, Cu, Dy, Er, Eu, Ga, Gd, Ge, Hf, Ho, In, La, Li, Lu, Mo, Nb, Nd, Ni, Pb, Pr, Rb, Sb, Sc, Sm, Sn, Sr, Ta, Tb, Th, Tl, Tm, U, V, W, Y, Yb, Zn, and Zr). Geochemical compositions were measured for multiple samples, and the results from each town were averaged into a single final value for each location. Due to differences in analysis between labs, sodium peroxide vs. tetraborate fusion, sample numbers for Cd and Li are fewer (19 PAS, 7 non-PAS, 3 unknown).

### Statistics and data analysis

The statistical analyses aimed to identify factors that differed between PAS and non-PAS samples. All statistical tests were conducted using SPSS 22.0 for Windows (Statistical Package for the Social Sciences, Inc., Chicago, Illinois, USA) with *α* = 0.05. All weight percentages were centered log-ratio (clr) transformed before statistical analysis (Grunsky, 2010; Pawlowsky-Glahn & Egozcue, 2012), where clr = ln(oxide weight%/geometric mean of composition). Following Nakagawa (2004), we chose not to adjust *α* for multiple tests. We further computed effect sizes, independent of sample size (in contrast to statistical significance) and more readily compared among different data sets and studies (Hojat & Xu, 2004; Nakagawa & Cuthill, 2007). For pairwise comparisons, we relied on Cohen's *d* using pooled standard deviation (Hojat & Xu, 2004), for which values of ~ 0.2, ~ 0.5, ≥ 0.8 are generally considered small, moderate, and large, respectively (Cohen, 1988). We computed multivariate eta-squared (*η*^2^) for DFA and *η*^2^ for ANOVA, with values of ~ 0.01, ~ 0.06, and ≥ 0.14 loosely regarded as small, moderate, and large, respectively (Cohen, 1988). Cohen's *d* provides a standardized unit of difference, whereas *η*^2^ indicates the approximate proportion of variance explained. Unless indicated otherwise, measures of central tendency presented are mean ± 1S.E. Individual models are specified in the results section. Parametric assumptions were not met for all analyses, and the following transformations were made: particle size analysis data < 10 µm was rank transformed, XRD bulk data was clr transformed, XRD clay data kaolinite was rank transformed, and smectite was natural log-transformed, and all major elements for geochemistry were clr transformed.

## Results

### Color analysis

**Fig. 2:**
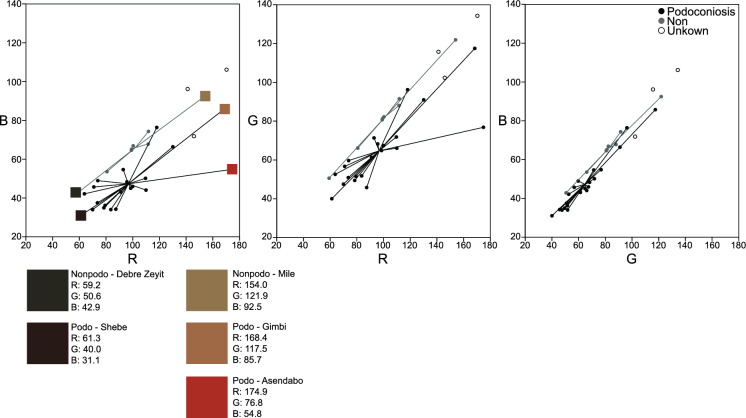
Scatterplots of sample values for a) Red vs Blue, b) Red vs Green, c) Green vs Blue (*n* = 32). Colored squares below graphs show sample locations with the highest red/green/blue and the lowest red/green/blue. R vs B shows the most separation between groups, while G vs B shows the least

Descriptive results for color show PAS were darker on average than the non-PAS (Table 2). An independent *t* test showed blue to be the only color channel of RGB significantly different between locations. Cohen's *d* results also indicate that blue has the largest effect size. The separation of the groups along the various channels can be seen in Fig. 2.

**Table 2:** Mean ± 1 SE of red, green, and blue colors for podoconiosis and non-podoconiosis associated soils (*n* = 29)

Color	Podo mean ± SE	Non-podo mean ± SE	*t* test *p *< 0.05	Cohen’s *d*
Red	96.71 ±6.71	101.99 ±9.63	0.660	− 0.19
Green	64.71 ±4.08	82.80 ±7.26	0.051	− 0.98
Blue	47.30 ±3.10	65.96 ±5.12	**0.008**	− 1.36

Independent *t* test scores and Cohen’s *d* effect sizes are also provided. Red is only slightly higher for podoconiosis cases than for non-podoconiosis cases and that it is blue that shows the most significant difference between groups. Bold indicates a statistically significant result

**Fig. 3:**
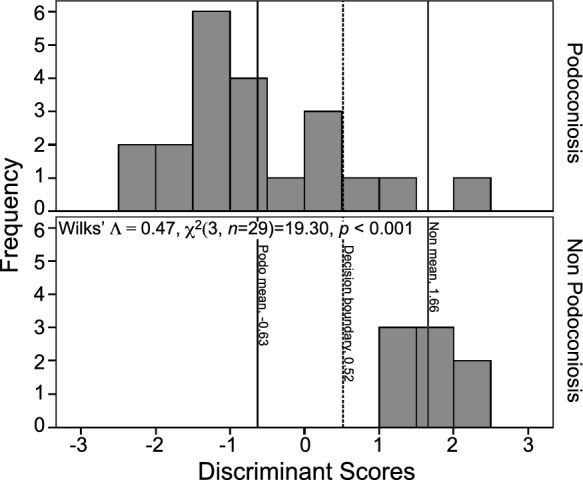
Histogram of discriminant function scores from color data (*n* = 29). Podoconiosis and non-podoconiosis means provided, as well as the decision boundary (DS = 0.31(B) – 0.19(G) – 0.01(R) – 1.93)

A two-way MANOVA reveals a significant difference between the two soil groups based on the linear combination of red, blue, and green (Wilks' ˄  = 0.47, *F*(3, 25) = 9.43, *p* < 0.001, $\eta$^2^ = 0.53). A follow-up discriminant function analysis (DFA) was conducted to determine whether the three variables—red, green, and blue—could predict the disease status of a soil (PAS or non-PAS). One significant function was generated, Wilks' ˄ = 0.47, *χ*^2^(3, *n* = 29) = 19.30, *p* < 0.001, indicating that the function of predictors significantly differentiates between the two soil groups. Standardized function coefficients find blue to make the largest unique contribution (4.42), then green (− 3.63), and red (− 0.34). The original classification reveals that 90.5% of PAS cases and 100% of non-PAS cases were correctly classified. For the overall sample, 93.1% were correctly classified. The means of the discriminant functions were consistent with these results. PAS had a function mean of − 0.63, while non-PAS had a mean of 1.66. DFA results were combined into the equation: DS = 0.31(B) − 0.19(G) − 0.01(R) − 1.93. This equation, in combination with Fig. 3, can be used to identify to which group a soil sample belongs. We used this predictor on samples collected from 3 towns of unknown disease status. One town was classified in the PAS group (BAB, − 0.57), and two were classified in the non-PAS group (BUL, 4.47; GOW, 3.76).

To identify a connection between soil color and a particular mineralogical agent, we conducted a regression of the blue channel against both the mineral and clay groups identified by XRD (details below in XRD section). A standard multiple regression was conducted to determine how the independent mineralogical variables (carbonate, clay, feldspar, mica, oxyhydroxides, pyroxenes, and quartz) could predict the blue color channel (*n* = 29). Regression results indicate that the overall model does not significantly predict blue (*R*^2^ = 0.32, *R*^2^_adj_ = 0.09, *F*_7,21_ = 1.38, *p* = 0.26).

**Fig. 4:**
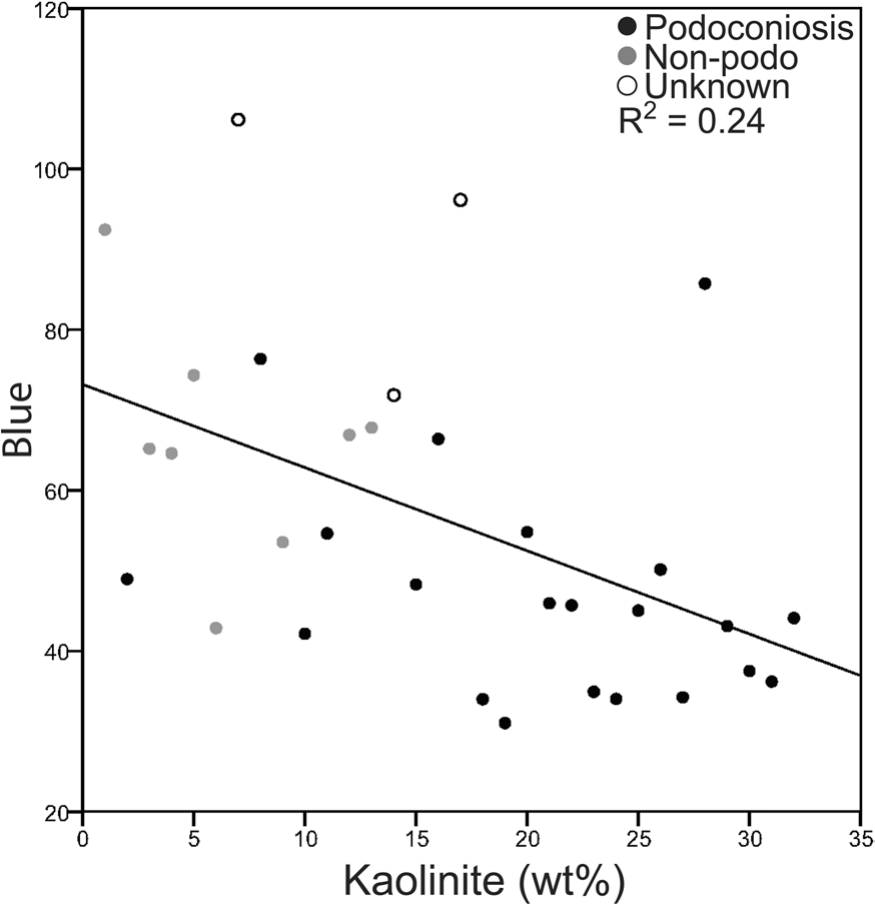
Scatterplot of rank transformed kaolinite vs the blue color (*n* = 32). Note the inverse relationship between the two variables: Blue = 101.90 – 1.662(rank(kaolinite))

A second multiple regression was conducted to determine whether the independent clay variables (dimica, kaolinite, smectite) could predict the blue color channel values (*n* = 21). The correlations of the variables included in the multiple regression are shown in Table 3. Regression results indicate the overall model significantly predicted blue (*R*^2^ = 0.33, *R*^2^_adj_ = 0.25, *F*_3,25_ = 4.13, *p* = 0.02), which accounts for 33% of the variance in blue. Regression coefficients indicate that kaolinite was the only variable that significantly contributed to the model (Fig. 4). Blue values were negatively associated with kaolinite, with coefficient *b* indicating a decrease of 1.66 units for every 1% increase in kaolinite (Table 3). Additionally, hue was calculated from the RGB values and a nonparametric Kruskal–Wallis run. No significance was found between PAS locations (13.90) and non-PAS locations (17.88; *χ*^2^(1) = 1.29, *p* = 0.26).

**Table 3:** Standard multiple regression results for blue color

	*b*	95% CI of *b*	*β*	*p*	Bivariate *r*	sr^2^
Dimica	− 0.33	− 0.81 to 0.15	− 0.35	0.169	0.08	0.07
Kaolinite	− 1.66	− 2.85 to − 0.47	− 0.98	**0.008**	− 0.52	0.25
Smectite	− 4.97	− 11.80 to 1.86	− 0.51	0.146	0.29	0.06

Kaolinite was rank transformed and smectite Ln transformed. Significant predictors indicated in bold. Kaolinite is the only significant predictor

*R*^2^ = 0.33, *R*^2^_adj_ = 0.25, *F*_3,25_ = 4.13, *p* = 0.02

sr^2^ is the squared partial correlation

### Particle size analysis

**Fig. 5:**
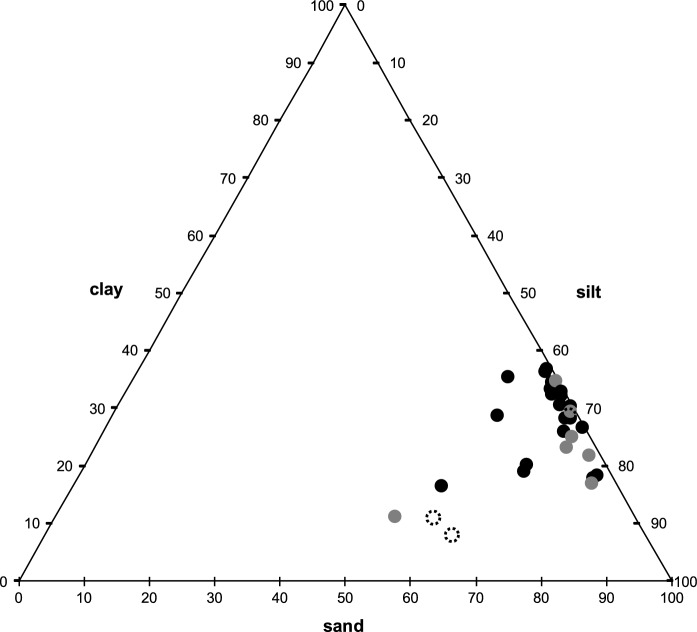
Soil texture diagram (*n* = 32). All samples fall into the clay category with no separation between podoconiosis and non-podoconiosis

**Fig. 6:**
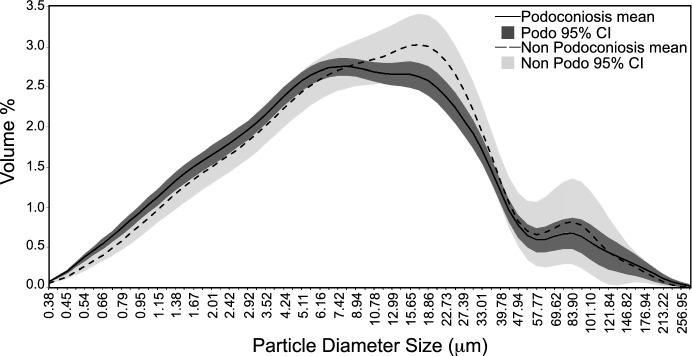
Particle size analysis results of particle size diameter by volume weight %, averaged for podoconiosis and non-podoconiosis samples (*n* = 29). Also included are 95% confidence intervals for each group. Podoconiosis samples on average are slightly higher in proportion to non-podoconiosis at diameters less than 10 µm, though this difference was not found to be statistically significant

**Fig. 7:**
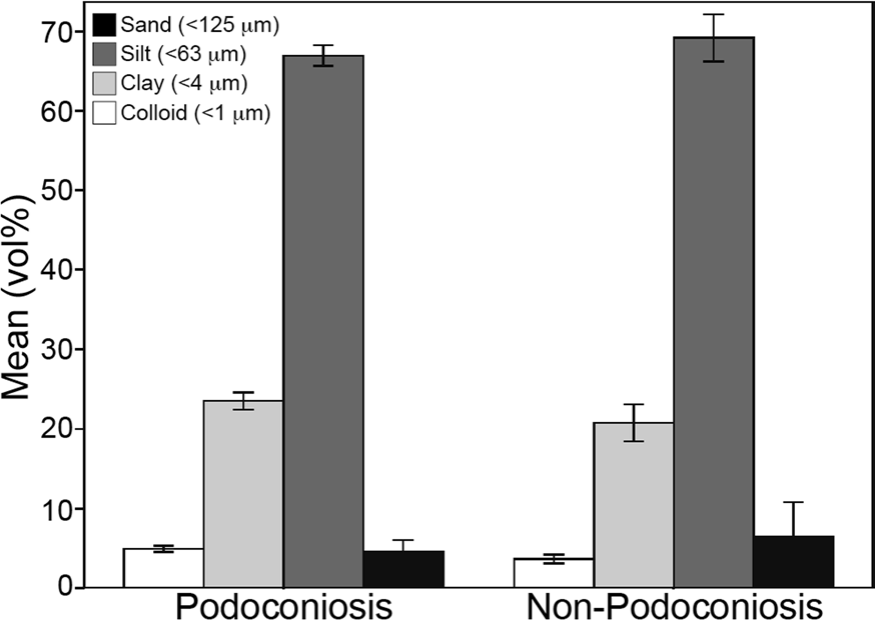
Histogram of particle size analysis data of grain size distribution (sand, silt, clay, colloid) for podoconiosis and non-podoconiosis samples (*n* = 29). Note the nearly identical results for the two groups

Descriptive data of clay, silt, and sand volume percent plotted on a ternary diagram show that all soil samples fell into the clay category, with no separation of the two groups (Fig. 5). Volume percent size distribution for both PAS and non-PAS locations also showed very similar average curves (Fig. 6). PAS samples had a slightly higher volume percent for approximately 8 µm and smaller, and non-PAS samples were slightly higher above the 8 µm size. A comparison of the mean volume percent of the four size classifications—weight % < 1.0 µm (colloid), 1–3.89 µm (clay), 3.9–62.9 µm (silt), and < 62.9 µm (sand)—between the two groups indicates only minor differences (Fig. 7).

Previous research had reported a higher proportion of clay and colloid size fraction in soils associated with podoconiosis, proposing this size fraction to be responsible for the onset of podoconiosis. To test these hypotheses, we ran two independent samples t-tests. The first compared the clay and colloid size fractions (< 4 µm), which reveals that the untransformed ratio PAS locations (28.09 ± 1.50) was not significantly different than for samples from non-PAS locations (24.16 ± 2.93; *t*(10.87) = 1.19, *p* = 0.64, Cohen’s *d* = 0.56). Our second *t* test compared the weight percent < 10 µm since this would be the size range easily engulfed by macrophages. These results also revealed no significant difference between PAS locations (16.05 ± 1.84) and non-PAS locations (12.25 ± 3.05; t(12.48) = 1.07, *p* = 0.306, Cohen’s *d* = 0.46).

A standard multiple regression was conducted to determine whether the independent variables (colloid, clay, silt) could predict the disease prevalence (*n* = 21). Regression results indicated the overall model did not significantly predict prevalence (*R*^2^ = 0.22, *R*^2^_adj_ = 0.08, *F*_3,17_ = 1.57, *p* = 0.23).

### Mineralogical analysis

#### Bulk mineralogy

Data for the identified minerals were combined into larger group classifications for analysis: carbonate, clay, feldspar, mica, oxyhydroxide, pyroxene, and quartz. The descriptive results of bulk data showed that PAS contained higher proportions of clay, pyroxene, oxides/hydroxides (hematite and magnetite), and quartz (Table 4). Comparison of effect size (Cohen's *d*) for the various mineral groups revealed pyroxenes to be the largest in contributing to group differences for our specific samples. A *t* test comparing the seven mineral groups showed a significant difference between PAS locations for only the pyroxene group (1.00 ± 0.99) and non-PAS locations (− 3.80 ± 1.81; *t*(11.44) = 2.33, *p* = 0.04, Cohen’s *d* = 1.06).

**Table 4:** Mean ±1 SE of mineral groups (untransformed) for podoconiosis and non-podoconiosis associated soils (*n* = 29)

Mineral group	Podo mean ± SE	Non-podo mean ± SE	*t* test *p *< 0.05	Cohen’s *d*
Carbonate	1 ± 1	4 ± 1	0.27	− 0.60
Clay	54 ± 6	54 ± 5	0.98	− 0.01
Feldspar	22 ± 5	23 ± 5	0.09	− 0.52
Mica	3 ± 2	6 ± 3	0.32	− 0.50
Ox/hydroxides	3 ± 1	2 ± 1	0.44	0.34
Pyroxene	3 ± 1	1 ± 1	**0.04**	1.06
Quartz	14 ± 2	11 ± 1	0.39	0.31

Independent *t* test scores and Cohen’s *d* effect sizes are also provided. Pyroxene is statistically significant between the groups and shows a larger effect size than any other mineral group. Bold indicates a statistically significant result

**Fig. 8:**
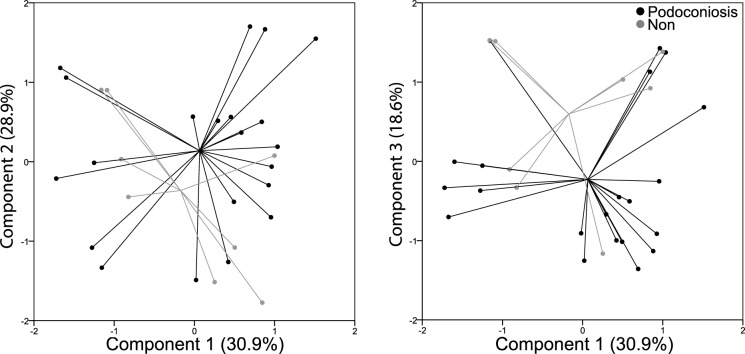
Principal component scores on Components 1 vs 2 and 1 vs 3 (centered log-ratio transformed data, *n* = 29). No separation of groups is visible along any of the 3 components

We employed PCA to investigate the latent structure in the set of clr-transformed weight percentages for the 7 mineral groups (clay, carbonate, feldspar, mica, oxyhydroxide, pyroxene, and quartz) that might relate to regions known to have podoconiosis. Principal component analysis was conducted using a varimax rotation. Eigenvalue and variance criteria (Mertler & Vannatta, 2005) indicated a three-component solution, with component 1 accounting for 30.9%, component 2 accounting for 28.9%, and component 3 accounting for 18.6% (Fig. 8). Table 5 presents the factor loadings for each component. The components provided no separation of the groups. MANOVA results indicated no significant difference between the two soil groups based on the linear combination of the three components identified by PCA (Wilks’ $\wedge$ = 0.80, *F*(3, 25) = 2.15, *p* = 0.12, *η*^2^ = 0.21).

**Table 5:** Factor loadings for varimax rotated components from principle component analysis of 7 mineral groups (centered log-ratio transformed, *n* = 29)

Mineral group	C1 30.9%	C2 28.9%	C3 18.6%
Mica	− 0.862		
Clay	0.831		
Quartz	0.805		
Oxyhydroxides		0.810	
Carbonate		− 0.807	
Feldspar		− 0.710	
Pyroxene			− 0.973

A standard multiple regression analysis evaluated how well the mineralogical groups (clay, carbonate, feldspar, mica, oxyhydroxide, pyroxene, quartz) predicted podoconiosis prevalence (*n* = 21). Regression results indicated that the overall model did not significantly predict prevalence (*R*^2^ = 0.38, *R*^2^_adj_ = 0.05, *F*_3,17_ = 1.15, *p* = 0.39).

#### Clay mineralogy

Mean values of kaolinite were higher in PAS samples than in the non-PAS samples (Table 6), while dimica and smectite were more abundant in non-podoconiosis samples. A *t* test comparing dimica, kaolinite, and smectite showed a significant difference between PAS and non-PAS locations for both kaolinite and smectite. Kaolinite showed the largest effect size of the three variables. We also found 8 samples from 5 locations contained low levels of gibbsite. We ratioed these gibbsite values to kaolinite and found a range of 1–7%. A MANOVA was conducted, and our results indicated a significant difference between the two soil groups based on the linear combination of dimica, kaolinite, and smectite (Wilks' $\wedge$ = 0.55, *F*(3, 25) = 6.86, *p* = 0.002, *η*^2^ = 0.45).

**Table 6:** Mean ± 1 SE of clay groups (kaolinite rank transformed, smectite Ln transformed) for podoconiosis and non-podoconiosis associated soils (*n* = 29)

Clay group	Podo mean ± SE	Non-podo mean ± SE	*t* test *p *< 0.05	Cohen’s *d*
Dimica	31 ± 3	41 ± 8	0.287	− 0.60
Kaolinite	55 ± 4	19 ± 4	**< 0.001**	2.00
Smectite	14 ± 4	40 ± 11	**0.028**	− 1.01

Independent *t* test scores and Cohen’s *d* effect sizes are also provided. Note that kaolinite is significantly higher in podoconiosis-associated soils and shows the largest effect size, while smectite is significantly higher in non-podoconiosis associated soils. Bold indicates a statistically significant result

**Fig. 9:**
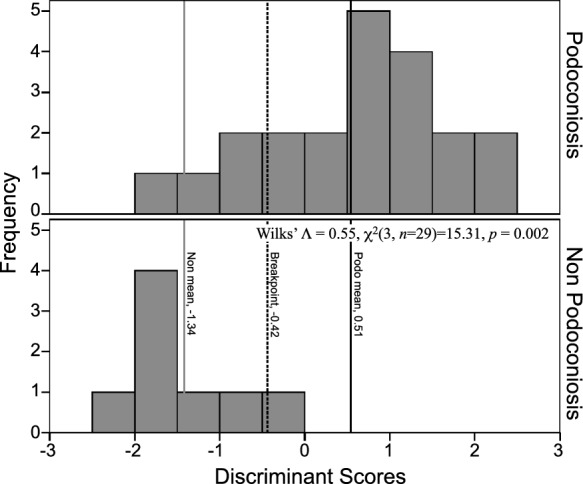
Histogram of discriminant function scores from analysis of clay groups (*n* = 29). Podoconiosis and non-podoconiosis means provided, as well as the decision boundary (DS = 0.000(dimica) + 0.154(rank(kaolinite)) + 0.148(Ln(smectite)) – 2.91)

A follow-up discriminant function analysis (DFA) determined whether the three variables—dimica, kaolinite, and smectite—could predict the disease status of a soil. One significant function was generated, Wilks' $\wedge$ = 0.55, *χ*^2^(3, *n* = 29) = 15.31, *p* = 0.002, indicating that the function of predictors significantly differentiates between the two soil groups. Standardized function coefficients showed that kaolinite makes the largest unique contribution (1.13), then smectite (0.23), followed by dimica (0.003). Original classification revealed that 90.5% of PAS cases and 75% of non-PAS cases were correctly classified. For the overall sample, 86.2% were correctly classified. Cross-validation derived 79.3% for the total sample. The means of the discriminant functions were consistent with these results, with a function mean of 0.54 for PAS and a mean of − 1.42 for non-PAS. DFA results were combined into the equation: DS = 0.000(dimica) + 0.154(rank(kaolinite)) + 0.148(Ln(smectite))—2.91. This equation, in combination with Fig. 9, can be used to identify to which group a soil sample belongs. We used it on samples collected from three towns of unknown disease status. All three towns were placed with podoconiosis (BAB, BUL, GOW).

A standard multiple regression analysis evaluated how well the clay groups (dimica, kaolinite, smectite) predict podoconiosis prevalence (*n* = 21). Regression results indicate that the overall model did not significantly predict prevalence (*R*^2^ = 0.28, *R*^2^_adj_ = 0.15, *F*_3,17_ = 2.19, *p* = 0.13).

#### Geochemistry

The analysis of ten major oxides showed that PAS had higher values of Al_2_O_3_, Fe_2_O_3_, MnO, P_2_O_5_, and TiO_2_ (see Table 7 for descriptive data, *t*-test, and Cohen's *d* results). An independent samples *t*-test revealed that transformed values for these elements in PAS were significantly different from those of non-PAS. In contrast, CaO, K_2_O, MgO, Na_2_O, and SiO_2_ were higher in non-PAS. Independent samples t-test results showed that transformed values for only CaO and Na_2_O were significant. Cohen's *d* shows Na_2_O, Al_2_O_3_, and Fe_2_O_3_ provided the largest effect sizes (listed in order of greatest to least size).

**Table 7:** Mean ± 1 SE of major oxides (centered log-ratio transformed) for podoconiosis and non-podoconiosis associated soils (*n* = 29)

Oxide	Podo mean ± S.E.	Non-podo mean ± S.E.	*t* test *p* < 0.05	Cohen’s *d*
Al_2_O_3_	22.61 ± 1.09	15.00 ± 0.92	**0.002**	1.75
CaO	1.29 ± 0.30	4.62 ± 1.55	**0.004**	− 1.37
Fe_2_O_3_	16.03 ± 1.08	8.90 ± 1.13	**< 0.001**	1.62
K_2_O	1.48 ± 0.17	2.74 ± 0.49	0.173	− 1.33
MgO	1.29 ± 0.17	2.24 ± 0.57	0.300	− 0.93
MnO	0.32 ± 0.02	0.24 ± 0.03	**0.026**	0.96
Na_2_O	0.76 ± 0.16	2.22 ± 0.18	**< 0.001**	− 2.20
P_2_O_5_	0.30 ± 0.03	0.20 ± 0.05	**0.002**	0.74
SiO_2_	53.17 ± 2.05	62.28 ± 3.14	0.965	− 1.02
TiO_2_	2.74 ± 0.24	1.57 ± 0.34	**< 0.001**	1.12

Independent *t* test scores and Cohen’s *d* effect sizes are also provided. Note the significantly higher values within podoconiosis-associated soils for Al_2_O_3_, Fe_2_O_3_, MnO, P_2_O_5_, and TiO^2^. Bold indicates a statistically significant result

**Fig. 10:**
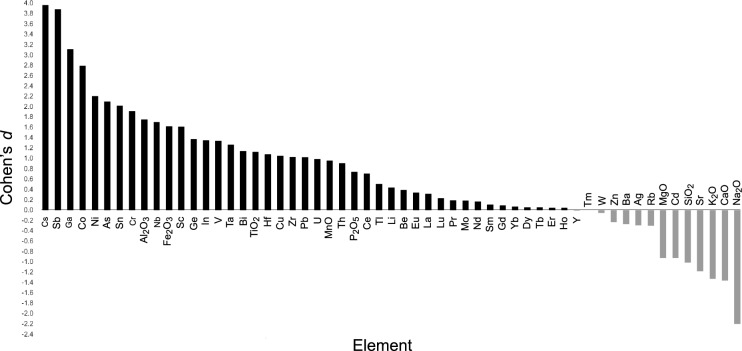
Cohen’s d effect sizes for all 57 elements analyzed. Positive values indicate a positive relationship with podoconiosis-associated soils and negative values indicate a positive correlation with non-podoconiosis associated soils

Descriptive data for the 47 trace elements found PAS had higher values, compared to non-PAS, for As, Be, Bi, Ce, Co, Cr, Cs, Cu, Ga, Ge, Hf, La, Li, Mo, Nb, Nd, Ni, Pb, Sb, Sc, Sn, Ta, Th, U, V, and Zr (see Table 8 for descriptive data, *t*-test, and Cohen's *d* results). Results from the independent samples *t *test found As, Bi, Co, Cr, Cs, Ga, Ni, Sb, and Sn significantly different between soil groups. Of elements with higher values for non-PAS, the independent samples *t*-test only found significance for Cd. Cohen's *d* (Fig. 10) shows Cs, Sb, Ga, Co, Ni, As, and Sn to provide the seven largest effect sizes (listed from greatest to least).

**Table 8:** Mean ± 1 SE of trace elements (untransformed) for podoconiosis and non-podoconiosis associated soils (*n* = 29)

Element	Podo mean ± S.E.	Non-podo mean ± S.E.	*t* test *p* < 0.05	Cohen’s *d*
Ag	0.78 ± 0.35	1.19 ± 0.29	0.041	− 0.30
As^+^	4.87 ± 0.58	0.21 ± 0.21	**< 0.001**	2.10
Ba	370.4 ± 34.10	412.14 ± 56.65	0.548	− 0.27
Be^+^	4.23 ± 0.54	3.29 ± 0.92	0.394	0.39
Bi^+^	0.16 ± 0.02	0.09 ± 0.02	**0.003**	1.14
Cd^+^	0.06 ± 0.03	0.17 ± 0.05	**0.010**	− 0.93
Ce	180.37 ± 20.05	145.64 ± 33.27	0.388	0.71
Co^+*^	37.11 ± 3.48	19.99 ± 3.89	**0.004**	2.79
Cr^+*^	145.12 ± 17.69	86.41 ± 19.37	**0.037**	1.91
Cs	2.76 ± 0.18	1.57 ± 0.19	**< 0.001**	3.96
Cu^+^	47.30 ± 5.34	33.54 ± 8.92	0.209	1.05
Dy	11.72 ± 1.16	11.52 ± 2.39	0.770	0.06
Er	6.71 ± 0.70	6.62 ± 1.36	0.835	0.05
Eu	2.71 ± 0.14	2.55 ± 0.33	0.665	0.34
Ga	31.83 ± 1.48	24.31 ± 1.50	**0.002**	3.11
Gd	11.66 ± 1.07	11.36 ± 2.26	0.721	0.09
Ge	2.29 ± 0.13	1.84 ± 0.22	0.112	1.37
Hf	15.47 ± 1.81	11.68 ± 2.29	0.253	1.08
Ho	2.32 ± 0.23	2.29 ± 0.47	0.810	0.04
In	0.07 ± 0.02	0.04 ± 0.08	0.288	1.35
La	72.24 ± 7.60	65.77 ± 13.96	0.691	0.32
Li	25.61 ± 2.59	23.13 ± 3.79	0.529	0.44
Lu	0.98 ± 0.10	0.92 ± 0.18	0.780	0.23
Mo^+*^	5.44 ± 1.10	0.92 ± 0.18	0.118	0.18
Nb	76.58 ± 10.24	50.41 ± 9.24	0.217	1.70
Nd	65.75 ± 6.48	62.58 ± 13.29	0.834	0.16
Ni^+*^	85.14 ± 10.51	48.54 ± 10.17	**0.021**	2.20
Pb^+^	23.52 ± 1.61	18.40 ± 3.48	0.211	1.02
Pr	17.28 ± 1.77	16.32 ± 3.55	0.812	0.19
Rb	71.77 ± 5.25	79.03 ± 16.81	0.690	− 0.31
Sb^+^	0.50 ± 0.08	0.19 ± 0.03	**0.002**	3.88
Sc	21.38 ± 2.05	13.26 ± 3.41	0.063	1.61
Sm	13.13 ± 1.24	12.72 ± 2.59	0.794	0.11
Sn^+^	7.13 ± 2.06	3.27 ± 0.62	**0.036**	2.02
Sr	111.64 ± 20.46	174.80 ± 36.21	0.126	− 1.19
Ta	5.15 ± 0.70	3.68 ± 0.72	0.231	1.26
Tb	1.92 ± 0.18	1.89 ± 0.38	0.772	0.05
Th^+^	14.16 ± 1.34	11.16 ± 2.24	0.273	0.91
Tl^+^	0.13 ± 0.03	0.10 ± 0.04	0.826	0.50
Tm	0.98 ± 0.11	0.98 ± 0.20	0.898	0.00
U	3.21 ± 0.26	2.35 ± 0.61	0.226	0.99
V^+^	208.46 ± 21.54	134.69 ± 37.53	0.114	1.34
W^+^	2.07 ± 0.26	2.14 ± 0.91	0.776	− 0.05
Y	59.19 ± 6.06	59.03 ± 11.78	0.874	0.01
Yb	6.38 ± 0.68	6.26 ± 1.24	0.880	0.07
Zn^+^	147.50 ± 12.30	155.51 ± 23.70	0.531	− 0.23
Zr	668.52 ± 81.82	503.39 ± 104.88	0.316	1.03

Independent *t* test scores and Cohen’s *d* effect sizes are also provided. Note the significantly higher values within podoconiosis-associated soils for As, Bi, Co, Cr, Cs, Ga, Ni, Sb, and Sn. Bold indicates a statistically significant result

*Above trigger action values

^+^Elements considered potentially harmful

**Fig. 11:**
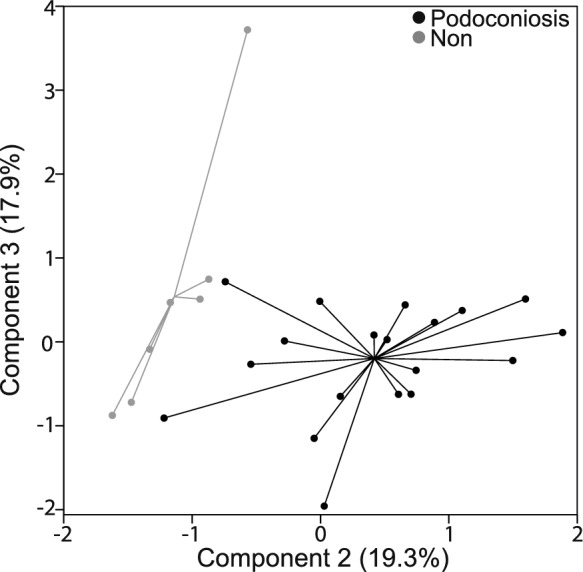
Principal component scores on Components 2 vs 3 (*n* = 29) for analysis of all 57 elements. Group separation is greatest along Component 2 (associated greatest to least with Al, Fe, Ca, Na, Ga, Ti, P, As, Cs, Mo, Bi, Sr, Mn, Ag)

We employed PCA to investigate the latent structure in the set of 57 elements that might relate to regions known to have podoconiosis. Principal component analysis was conducted using a varimax rotation. Eigenvalue and variance criteria (Mertler & Vannatta, 2005) indicated a six-component solution (component loadings and variance provided in Table 9). Component 2 alone showed a clear separation from all other components (Fig. 11).

**Table 9:** Factor loadings for varimax rotated components from principle component analysis of 57 elements (*n* = 29)

C1—39.3%		C2—19.3%		C3—17.9%		C4—5.3%		C5—5.2%		C6—4.1%	
Element	CS	Element	CS	Element	CS	Element	CS	Element	CS	Element	CS
Tb	0.960	Al_2_O_3_	0.937	Rb	0.911	Sn	0.804	Li	0.766	W	0.735
Gd	0.960	Fe_2_O_3_	0.916	K_2_O	0.858	Sb	0.735	Ni	0.614		
Dy	0.956	CaO	− 0.903	SiO_2_	0.817	Tl	0.730				
Sm	0.953	Na_2_O	− 0.828	V	− 0.738						
Ho	0.952	Ga	0.803	Pb	0.722						
Y	0.946	TiO_2_	0.796	Th	0.708						
Er	0.944	P_2_O_5_	0.754	U	0.706						
Nd	0.943	As	0.749	Cu	− 0.706						
Tm	0.935	Cs	0.705	Co	− 0.705						
Pr	0.929	Mo	0.642	Sc	− 0.697						
Yb	0.926	Bi	0.639	MgO	− 0.693						
Lu	0.919	Sr	− 0.620	Cr	− 0.578						
La	0.894	MnO	0.620								
Eu	0.887	Ag	− 0.518								
Zn	0.853										
Ce	0.850										
Zr	0.798										
Be	0.786										
Hf	0.782										
Ge	0.762										
Ba	− 0.744										
Ta	0.727										
In	0.722										
Nb	0.654										
Cd	0.601

**Fig. 12:**
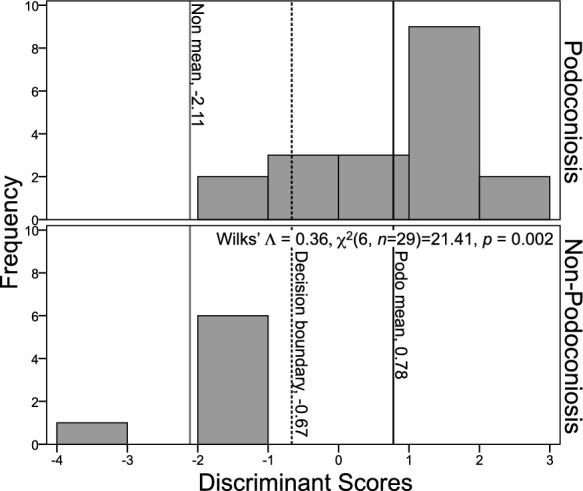
Histogram of discriminant function scores of the six PCA components from analysis of all 57 elements (*n* = 29). Podoconiosis and non-podoconiosis means are provided, as well as the decision boundary (DS = 0.17(C1) + 1.44(C2) − 0.68(C3) + 0.30(C4) + 0.09(C5) + 0.04(C6))

We conducted a follow-up discriminant function analysis (DFA) to determine whether the six PCA components could predict the disease status of a soil (PAS or non-PAS). One significant function was generated, Wilks' $\wedge$ = 0.36, *χ*^2^(6, *n* = 29) = 21.41, *p* = 0.002, indicating that the function of predictors significantly differentiated between the two soil groups. Standardized function coefficients show component 2 made the largest unique contribution (1.04) followed by component 3 (− 0.65). The original classification reveals 89.5% of PAS cases and 100% of non-PAS cases were correctly classified. For the overall sample, 92.3% were correctly classified. Cross-validation derived 88.5% for the total sample. The means of the discriminant functions were consistent with these results, with a function mean of 0.78 for PAS and a mean of − 2.11 for non-PAS. DFA results were combined into the equation: DS = 0.17(C1) + 1.44(C2) − 0.68(C3) + 0.30(C4) + 0.09(C5) + 0.04(C6) (Fig. 12).

**Fig. 13:**
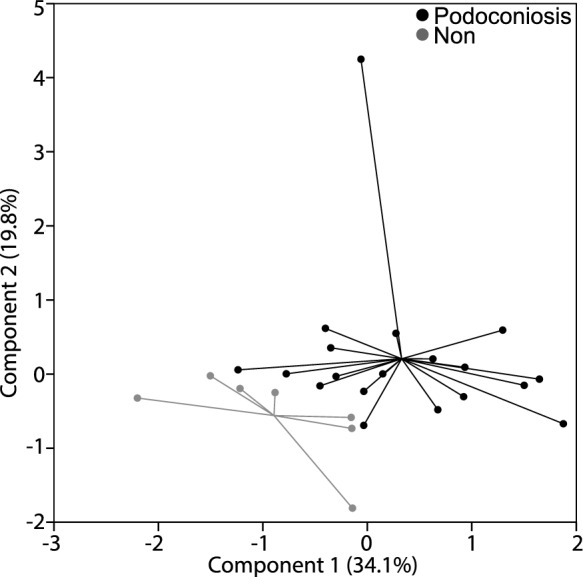
Principal component scores on Components 1 vs 2 (*n* = 29) for analysis of potentially harmful elements. The majority of separation of groups is along Component 1 with only minimal separation along Component 2

We employed PCA to investigate the latent structure in the set of 17 elements that might relate to PAS regions. Principal component analysis was conducted using a varimax rotation. Eigenvalue and variance criteria (Mertler & Vannatta, 2005) indicated a four-component solution (component loadings and variance provided in Table 10). Component 1 showed the clearest separation from all other components (Fig. 13).

**Table 10:** Factor loadings for varimax rotated components from principle component analysis of 17 potentially harmful elements (*n* = 29)

Element	C1, 34.1%	C2, 19.8%	C3, 17.2%	C4, 12.5%
Co	0.948			
Cu	0.926			
Ni	0.904			
Cr	0.888			
V	0.869			
As	0.590			
Sn		0.910		
Sb		0.892		
Tl		0.574		
Bi			0.791	
W			0.697	
Mo			0.640	
Th			0.578	
Pb	− 0.573	0.550	0.558	
Zn				0.898
Cd				0.654
Be				0.552

**Fig. 14:**
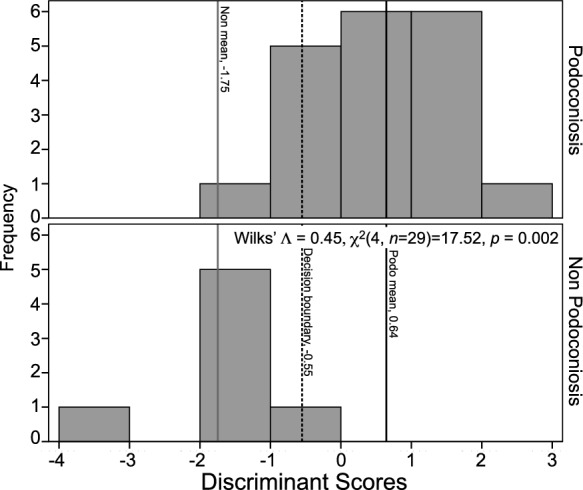
Histogram of discriminant function scores of four PCA components from analysis of 17 potentially harmful elements (*n* = 29). Podoconiosis and non-podoconiosis means provided, as well as the decision boundary (DS = 1.09(C1) + 0.68(C2) + 0.68(C3) + 0.11(C4))

We conducted a follow-up discriminant function analysis (DFA) to determine whether the four PCA components could predict the disease status of a soil (PAS or non-PAS). One significant function was generated, Wilks' $\wedge$ = 0.45, *χ*^2^(4, *n* = 29) = 17.52, *p* = 0.002. It indicated that the function of predictors significantly differentiated between the two soil groups. Standardized function coefficients found component 1 made the largest unique contribution (0.93), components 2 and 3 tied for second (0.65), and component 4 made the least contribution (0.11). Original classification revealed 94.7% of PAS cases and 85.7% of non-PAS cases were correctly classified. For the overall sample, 92.3% were correctly classified. Cross-validation derived 88.5% for the total sample. The means of the discriminant functions were consistent with these results, with a function mean of 0.64 for PAS and a mean of − 1.75 for non-PAS. DFA results were combined into the equation: DS = 1.09(C1) + 0.68(C2) + 0.68(C3) + 0.11(C4) (Fig. 14).

**Fig. 15:**
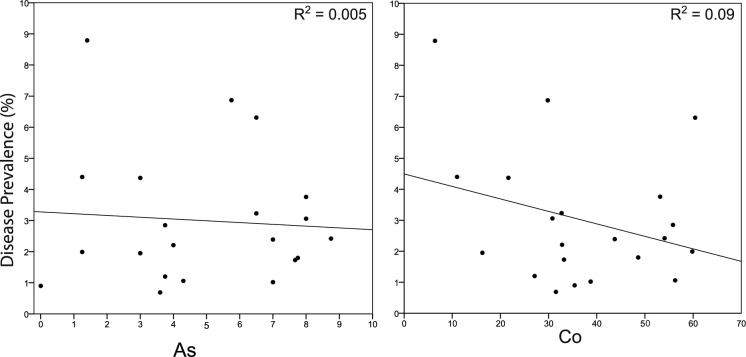
Histogram of discriminant function scores of four PCA components from analysis of 17 potentially harmful elements (*n* = 29). Podoconiosis and non-podoconiosis means provided, as well as the decision boundary (DS = 1.09(C1) + 0.68(C2) + 0.68(C3) + 0.11(C4))

A standard multiple regression was conducted to determine whether the elements that comprise PCA component 1 (As, Co, Cr, Cu, Ni, V) could predict the disease prevalence (*n* = 21). The correlations of the variables included in the multiple regression are shown in Table 11. Regression results indicate the overall model significantly predicted disease prevalence (*R*^2^ = 0.56, *R*^2^_adj_ = 0.36, *F*_6,14_ = 2.90, *p* = 0.047), which accounted for 56% of the variance in prevalence. Regression coefficients indicated that both Co and Ni significantly contributed to the model (Fig. 15). Disease prevalence was negatively associated with Co, with b indicating a decrease of 0.2% in prevalence for every 1 ppm increase in Co. In contrast, disease prevalence was positively correlated with Ni, with b indicating an increase of 0.1% in prevalence for every 1 ppm Ni.

**Table 11:** Standard multiple regression results of disease prevalence (*n* = 21)

	*B*	95% CI of *b*	*β*	*p*	Bivariate *r*	sr^2^
Tab	− 0.25	− 0.70 to 0.20	− 0.31	0.260	− 0.07	0.09
Co	− 0.21	− 0.37 to − 0.06	− 1.60	**0.008**	− 0.30	0.39
Cr	− 0.01	− 0.04 to 0.02	− 0.33	0.517	0.01	0.03
Cu	− 0.05	− 0.15 to 0.05	− 0.61	0.273	− 0.12	0.09
Ni	0.09	0.02 to 0.16	2.01	**0.014**	0.08	0.36
V	0.01	− 0.01 to 0.04	0.61	0.212	− 0.29	0.11

Co and Ni are both significant

Significant predictors indicated in bold

## Discussion

A causal relation between local soil and podoconiosis has long been postulated but little understood. Over the decades, a handful of geology-specific studies have been done. Still, the health problems associated with podoconiosis have led to more emphasis on the disease's medical or public health component. We believe that without a clear knowledge of the causal agent, we will only be able to treat the symptoms rather than work on possible eradication or mitigation before the process starts. The research presented here has been undertaken to provide data that can help fill gaps in the geological side of the podoconiosis story. We propose four key results from our data that can help focus future podoconiosis research.

### Podoconiosis-associated soils are much more highly weathered, leading to enrichment of insoluble minerals and elements

PAS has long been associated with volcanic rocks (Crivelli, 1986; Price, 1976; Price & Bailey, 1984). Cooper et al. (2019) proposed the bedrock to be a unique alkaline- and silicon-rich geochemistry, particularly associated with incompatible elements. Additionally, the correlation of PAS soils with high rainfall has been well established. The first mention of the association is by Price (1974), who suggested an isohyet of 1000 mm of rainfall annually. Work by Deribe et al. (2015) found the same isohyet to be one of the strongest predictors for the occurrence of podoconiosis. However, whether the relationship between the rainfall and the disease was one of correlation or true causal mechanisms has remained unclear. Analysis of data from this study suggests that the unique alkaline bedrock combined with higher amounts of rainfall fits a known weathering model and is a key component in the podoconiosis story.

Our data strongly indicate that PAS are more weathered than non-PAS. Several lines of evidence support this, including both mineralogy and geochemistry. Bulk mineralogy shows PAS to have elevated amounts of clays, pyroxenes, oxyhydroxides, and quartz. These particular minerals are composed chiefly of elements with a z/r between 4 and 8, which are less soluble, enriching their concentrations within soils that are more highly weathered (Railsback, 2003). Geochemical results show significantly higher amounts of Al, Fe, Mn, P, and Ti in PAS while at the same time showing significantly lower quantities of Ca and Na, once again indicating that the more soluble elements have been removed and enrichment of the insoluble fraction has occurred. Additional support for a weathering model comes from clay analyses. Several PAS samples contain 1 to 7% gibbsite when ratioed to kaolinite. Stability plots of typical feldspar clay assemblages show increasing water, and H^+^ moves stability from feldspar through kaolinite to gibbsite (Steinmann et al., 1994). These results mirror the Le Blond et al. (2015) paper that compared the soils from the upper part of the Mt Choke shield volcano to soils formed from the flood basalts at its base. These two soil types can be interpreted as proxies for non-PAS soils and PAS, respectively. Their results found that the geochemistry associated with soils formed from the flood basalts at the base showed a higher extent of weathering than the soils from higher on the volcano. An additional connection that can be tied to increased weathering was reported by Gislam et al. (2020). Their results indicated an increased level of Be in samples associated with podoconiosis. They suggest that the Be is perhaps concentrated as it is bound to clay minerals which would occur during weathering.

We propose that when taken together, these lines of evidence lead to a geological model of fate and transport. Alkaline volcanic bedrock across Ethiopia undergoes a wide variety of weathering, with higher elevation areas receiving more rainfall. Differences in weathering lead to more extensive washing of the PAS, enrichment in insoluble elements, and a shift in the mineral assemblage.

### Kaolinite or gibbsite may play a more significant role in the etiology of podoconiosis than previously recognized

Clarification is needed between two different hypotheses regarding clays in podoconiosis. For decades, some soil component has been identified as a causal factor in podoconiosis. Whether due to their large surface area to size ratio or because their small size allows easier access into the body, clay-sized particles are primary candidates as harmful agents. Price and Plant (1990) expressed their desire for further study: "If a greater number of results were available, it should be possible to establish levels of particle size/frequency to match the prevalence of the disease, and so establish a risk factor to which residents in a given area would be exposed." Previous quantitative data by Price and his colleagues (Price & Bailey, 1984; Price & Plant, 1990; Price et al., 1981) reported higher amounts of clay and colloid-size particles in PAS. Important to note that Price used a different classification scale than we did, citing 2–10 µm as fine silt and < 2 µm as clay rather than the 1–4 µm for clay given by the classic Wentworth scale. Molla et al. (2014) report that their multivariate model did not find particle size significantly associated with disease prevalence. Results of a quantitative study by Le Blond et al. (2017) report a significant difference between soil groups when using de-flocculent and no significant difference when using water. Our data indicate no significant difference in particle size between our two soil groups. However, the caveat must be made that while no significant difference was found in colloid size fraction either, our measurements were made on an instrument that is less precise for sizes that small. Particle size analysis algorithms are based on spherical geometry (Stokes equation), and phyllosilicates, such as kaolinite, are composed of layers that typically weather into plate-like particles that maintain a large hydration sphere. Particle morphology investigated by Le Blond et al. (2017) found this to be the case, reporting a variety of shapes and clusters. Without a way to correct this difference between theoretical and actual, measurements may return larger size estimations than are truly characteristic of the soils. We are, however, not alone in our conclusions. Molla et al. (2014) also reported that particle size alone is not significantly associated with disease prevalence.

The second hypothesis suggests greater amounts of mineralogical clay are found in PAS. Two papers quantified differences in the amount of clay between podoconiosis-associated soils and non-associated soils, and both report twice the clay content in disease-associated soils (Price & Bailey, 1984; Price & Henderson, 1981). Historically, kaolinite has been the most frequently associated with PAS, with Price seeming to place considerable importance on it (Price, 1976, 1988). Illite and smectite have also been reported (Molla et al., 2014; Price et al., 1981). Interestingly, Molla et al. (2014) found smectite to be the most important predictor for podoconiosis prevalence, reporting a tripling in the disease case count for every 1% increase in the clay. They suggest that the high adherence of smectites to the skin might increase transdermal uptake of potential toxins. Despite the previous mention by other papers of high amounts of kaolinite, Molla et al. (2014) found no significant disease association. Our results contradict the reports of higher amounts of clay, as we found no significant difference in overall clay content between the two soil groups. As for clay species, we found PAS to have nearly three times the amount of kaolinite than non-PAS.

We also found smectite to be nearly three times more abundant in non-PAS than in PAS. It is unclear at this time why our results differ so dramatically from those of Molla's group. Le Blond et al. (2017) report a moderate correlation between the soil's proportion of smectite and kaolinite and the hemolytic potential. We believe this toxicity is due to clays' adsorbent properties and discuss this below in the next section. A final caveat must be made regarding the sheer complexity of the phyllosilicates. Our study was only able to assign to general categories however, there is a wealth of further data that could be collected related to composition, size, interlayering, stacking, or other characteristics that may give a more detailed correlation with the effects of the clay polytypes.

Related to clay minerals is the ongoing discussion regarding silicon and aluminum associated with podoconiosis. The origin of a connection between silica, aluminum, and podoconiosis is from a study by Price (1972) which reports finding "compounds of aluminum and silica" within lymph nodes of affected individuals. Further studies continued to report the presence of these elements, either alone or as a ratio of the two (Blundell et al., 1989; Heather & Price, 1972; Price & Henderson, 1978, 1979; Price & Pitwell, 1973; Price et al., 1981). Current research continues to investigate this connection. Molla et al. (2014) reported quartz (crystalline silica) as a predictive variable in their final model. Le Blond et al. (2017) found that an increase in Al_2_O_3_ was related to an increase in hemolytic activity. Gislam et al. (2020) identified quartz as significant in their PCA, showing separation between endemic and non-endemic groups. From a geological perspective, these results remain murky due to the lack of clarity regarding the form of silica. Conflation of the mineral quartz with the more generic term of silica has led to a seemingly general misinterpretation that the presence of silicon in any form must be toxic. Our bulk soil samples' results using XRD show that quartz abundance is higher in PAS. However, the *t*-test and Cohen's *d* effect size results indicate no significant difference between the two groups. We argue that neither the minerals containing silicon nor the aluminum are toxic and that the higher prevalence of silicon and aluminum within PAS is merely a reflection of the higher prevalence of more weathered clays, such as kaolinite and gibbsite. As the Ca, K, Mg, and Na, present in feldspars, ferromagnesium minerals, smectite and illite are hydrolyzed, Al and Si become more prominent in the soils. We propose the kaolinite and gibbsite themselves are key, either due to their higher gross proportion within the soils or due to higher surface area, as a vector for potentially harmful trace elements.

### Future medical research should focus on As, Co, Cr, Cs, Mo, and Ni

Clay minerals within soils and sediments are some of the most important adsorbents for metals and oxyanions within natural systems (Sparks, 2005). The propensity of clays to adsorb trace elements is not a new concept within podoconiosis, as mentioned by Price early in his research (Price, 1974). Coming from a geological bias, the ubiquitous nature of all major elements, including Al and Si, led us to discount their disease-causing effect as ions. We hypothesize that if a geological control of some ion exists, it must act on the trace elements. Previous podoconiosis research has identified a variety of trace elements, including Ag, Ba (2), Be (2), Bo, Ca (3), Ce, Co, Cr (3), Cu, Fe (5), In, K (2), La, Li, Mg (3), Ni (3), Pb, Rb, S, Sb, Sc, Sr (2), Ta, Ti (3), Tl, V, Yt, Zn, and Zr (times cited) (Gislam et al., 2020; Price, 1974; Price & Henderson, 1979, 1981; Price & Pitwell, 1973; Spooner & Davies, 1986). The oxides of many of these elements, including Be, Cr, Fe, In, La, Mg, Ni, Pb, Ta, Ti, V, Zn, and Zr, are relatively stable and concentrate in soil (Railsback, 2003). However, a lack of quantification in addition to small sample sizes, has provided little quantitative support. Thus, one of the main goals of our research was to analyze trace elements identified as potentially harmful to humans to determine whether any of them stand out. Our analyses, which included 57 trace elements, indicate As, Co, Cs, and Ni are significantly higher in PAS and have large effect sizes. Additionally, when analyzed using PCA, As, Cs, and Mo load onto component 2, which provides the most separation between the two soil groups. When analyses involving only potentially harmful elements were run, these same elements were again prominent. Mean values of Co, Cr, Mo, and Ni show all four above trigger action values set by many countries (Simon, 2014). Principle component analyses found As, Co, Cr, Cu, Ni, and V to load onto component 1, which provides the largest separation between the two soil groups. That both Co and Ni are significant predictors in our regression model further strengthens the argument. While Co is found in significantly higher proportions in PAS, the regression model shows that it has an inverse relationship with prevalence. We believe that this suggests a potential dose–response of the disease to Co. Thus, based on their repeated prominence, we recommend As, Co, Cr, Cs, Mo, and Ni become high priority research targets for future medical research related to podoconiosis.

### Soil color can provide a fast, inexpensive, and reliable method for identifying soils of concern for inducing podoconiosis

Terms, such as "tropical red soil" and "red-clay soil", have persisted throughout literature related to podoconiosis (Davey & Newport, 2007; Molla et al., 2014; Price, 1974; Wanji et al., 2021). Weathering of both basaltic and alkaline volcanic rocks has been proposed as sources for these red clays (Le Blond et al., 2015; Price, 1976; Price & Bailey, 1984). Despite the frequent mention, however, no quantitative data existed on whether the soil color is significantly different in areas of podoconiosis. Our multivariate analysis of the red, green, and blue color channels show they can discriminate between the two soil groups. Unexpectedly, it is significant for the blue channel alone and not the red channel we had hypothesized. Our data revealed an inverse relationship between the blue color of a sample and the amount of kaolinite. Using a DFA, we constructed an equation based on our samples that provides a decision boundary to classify soils by color. This observation may allow a simple field test to differentiate between the two groups using a hand-held color analyzer or possibly more globally using remote sensing. While podoconiosis continues to be a complicated disease, the ability to identify toxic soils based on their color could be a beneficial tool for researchers, healthcare workers, and public health planning. Though our results are specific to Ethiopia, we believe the methodology is reproducible in other countries.

## Conclusions

Podoconiosis is a complex disease that spans a large geographical area, and "diverse research groups are needed to accelerate the elimination of podoconiosis". Effort is needed to help focus research so that time and money can be used as efficiently as possible. We believe our study has added significantly to the amount of data available; however, more work still needs to be done to test a range of hypotheses for the etiology of podoconiosis. Our results indicate podoconiosis-associated soils are more highly weathered than non-podoconiosis associated soils. Enrichment of kaolinite and gibbsite suggests that these minerals, their surface chemistry, or trace elements associated with them should be prioritized in future podoconiosis research. Specific trace elements enriched in PAS soils are As, Co, Cr, Cs, Mo, and Ni. Soil color may be an efficient tool in identifying agricultural fields at greater risk for inducing podoconiosis. If problem soils can be identified, educational efforts and preventative measures can focus on areas of greatest need. We are hopeful that with a greater understanding of the geological associations with PAS, remediation of the soils might decrease the affected populations.
